# The role of zinc transporter proteins as predictive and prognostic biomarkers of hepatocellular cancer

**DOI:** 10.7717/peerj.12314

**Published:** 2021-10-15

**Authors:** Ceylan V. Bitirim

**Affiliations:** Stem Cell Institute, Ankara University, Ankara, Turkey

**Keywords:** Zinc transporters, Hepatocellular carcinoma, Zinc, Tumour grade

## Abstract

Identification of the key processes involved in the tumor progression, malignancy and the molecular factors which are responsible for the transition of the cirrhotic cells to the tumor cells, contribute to the detection of biomarkers for diagnosis of hepatocellular carcinoma (HCC) at an early stage. According to clinical data, HCC is mostly characterized by a significant decrease in zinc levels. It is strongly implied that zinc deficiency is the major event required in the early stages of tumor formation and development of malignancy. Due to this reason, the definition of the molecular players which have a role in zinc homeostasis and cellular zinc level could give us a clue about the transition state of the cirrhosis to hepatic tumor formation. Despite the well-known implications of zinc in the development of HCCthe correlation of the expression of zinc transporter proteins with tumor progression and malignancy remain largely unknown. In the present study, we evaluated in detail the relationship of zinc deficiency on the prognosis of early HCC patients. In this study, we aimed to test the potential zinc transporters which contribute tothe transformation of cirrhosis to HCCand the progression of HCC. Among the 24 zinc transporter proteins, the proteins to be examined were chosen by using Gene Expression Profiling Interactive Analysis (GEPIA) webpage and RNA-seq analysis using TCGA database. ZIP14 and ZIP5 transporters were found as common differentially expressed genes from both bioinformatic analyses. ZnT1, ZnT7 and ZIP7 transporters have been associated with tumor progression. Relative abundance of ZnT1, ZIP5 and ZIP14 protein level was determined by immunohistochemistry (IHC) in surgically resected liver specimens from 16 HCC patients at different stages. IHC staining intensity was analyzed by using ImageJ software and scored with the histological scoring (H-score) method. The staining of ZnT1 was significantly higher in Grade III comparing to Grade II and Grade I. On the contrary, ZIP14 staining decreased almost 10-foldcomparing to Grade Iand Grade II. ZIP5 staining was detected almost 2-fold higher in cirrhosis than HCC. But ZnT1 staining was observed almost 3-fold lower in cirrhosis comparing to HCC. Intracellular free zinc level was measured by flow cytometry in Hep40 and Snu398 cells using FluoZin-3 dye. The intracellular free zinc level was almost 9-fold decreased in poor differentiated Snu398 HCC cells comparing to well differentiated Hep40 HCC cells.This report establishes for the first time the correlation between the expression pattern of ZIP14, ZnT1 and ZIP5 and significant zinc deficiency which occurs concurrently with the advancing of malignancy. Our results provide new molecular insight into ZnT1, ZIP14 and ZIP5 mediated regulation of cellular zinc homeostasis and indicate that zinc transporters might be important factors and events in HCC malignancy, which can lead to the development of early biomarkers.

## Introduction

HCC is recognized as the third leading cancer with a high mortality rate and the incidence level continues to increase worldwide (Siegel, Naishadham & Jemal, 2012). Despite the studies carried on to find out the fundamental etiologic factors and mechanisms, the major processes which are involved in the development of HCC remain largely unknown and speculative. Identification of the key processes involved in the tumor progression and malignancy and the molecular factors which are responsible for the transition of the cirrhotic cells to the tumor cells, contribute to the detection of biomarkers for diagnosis of HCC at an early stage. Instead of innumerable studies, the definition of molecular signature is very difficult due to the highly heterogeneous genetic alteration profiles of HCC tumors (Costello & Franklin, 2014; Qian et al., 2021).

Zinc is a trace element that is involved in a broad spectrum of biological processes through the binding to a wide range of enzymes and transcription factors. Liver is the primer organ responsible for zinc metabolism which can be affected by liver diseases (Patel & Bansal, 2021). Previous studies have shown that zinc concentration in the cancerous liver tissue is significantly lower (almost 55% decrease) than in noncancerous liver tissues (Lichten & Cousins, 2009; To et al., 2020). Moreover, according to clinical data, HCC is mostly characterized by a significant decrease in zinc levels (Costello & Franklin, 2014). Taken together, these results strongly imply that zinc deficiency is the major event required in the early stages of tumor formation and development of malignancy in liver.

The evolution of the chronic degenerative disease is characterized by progression through intermediate states to advanced disease and death, such as liver cirrhosis that can evolve to hepatocellular carcinoma (Omran et al., 2017). Hence, liver cirrhosis represents a common outcome of chronic liver injuries such as immune dysfunction, impaired thyroid hormone and protein (Portolani et al., 2006; Grüngreiff, Reinhold & Wedemeyer, 2016). Due to this reason, the definition of the molecular players which have a role in zinc homeostasis and cellular zinc level could give us a clue about the transition state of the cirrhosis to hepatic tumor formation. 

**Figure 1:**
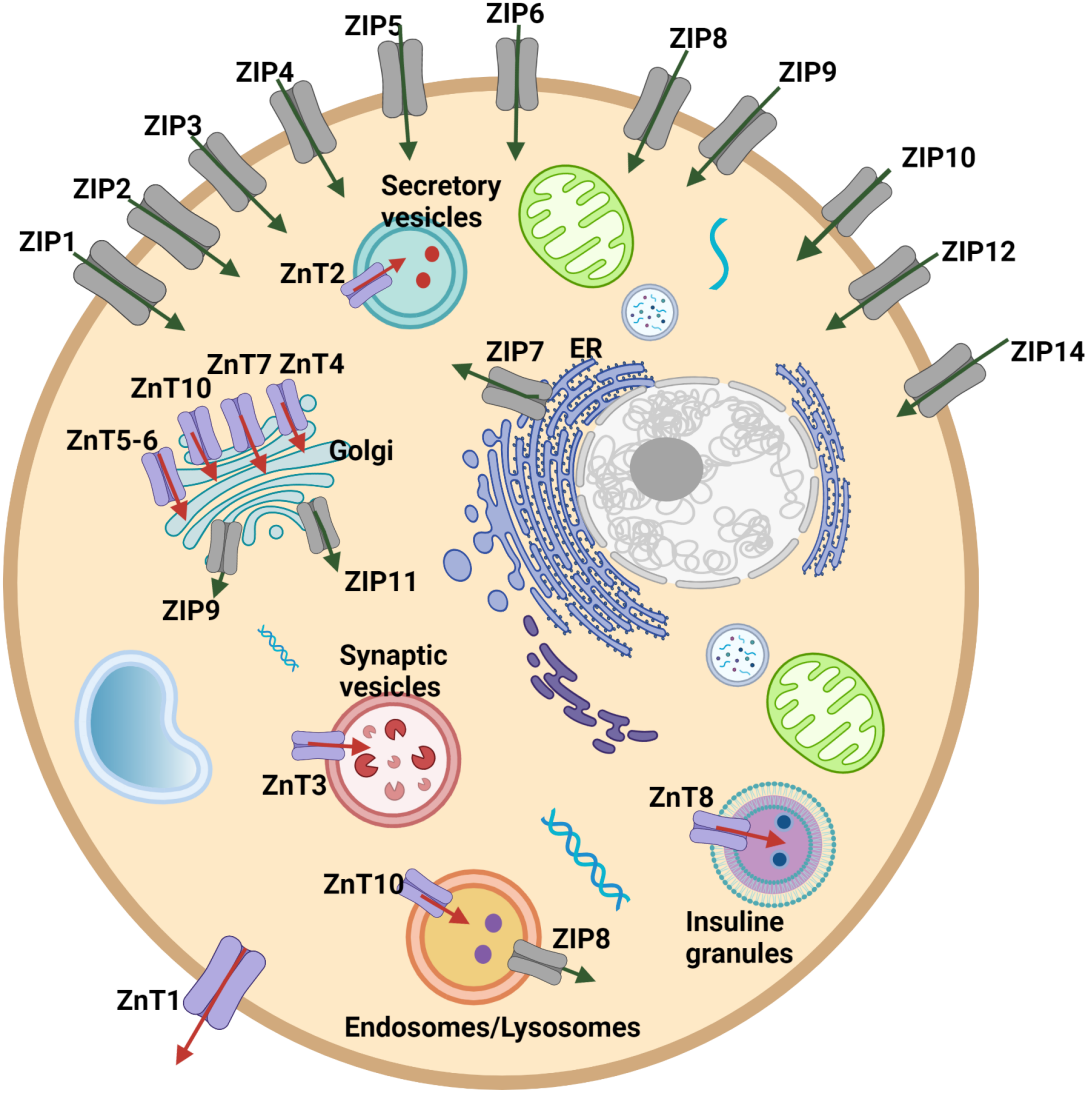
Cellular and subcellular localization of zinc transporter proteins. Red arrows indicate zinc efflux from the cytoplasm to the extracellular side or vesicles/organelles. Green arrows indicate zinc influx from extracellular space or intracellular vesicles into the cytoplasm.

Cellular and systemic zinc homeostasis is regulated by zinc transporter proteins. Up to date, 10 zinc transporters (ZnT, SLC30 family), mediate the zinc efflux from the cytoplasm to the extracellular side or vesicles/organelles, and 14 ZIP transporters (ZIP, SLC39 family), which provide the increase in the intracellular zinc level by accelerating the influx of zinc from extracellular space or intracellular vesicles into the cytoplasm, have been identified in human cells (Fig. 1). Zinc transporters have a specific tissue expression with different responsibilities and work coordinately to regulate intracellular, extracellular, or organelle-based translocation of zinc ions (Lichten & Cousins, 2009). Despite the well-known implications of zinc in the development of HCC, the correlation of the expression of zinc transporter proteins with tumor progression and malignancy remains largely unknown. The correlation between the ZIP14 downregulation and depletion of zinc in the hepatoma cells was reported for the first time by Costello & Franklin (2014). However, the alteration of the expression pattern of ZIP14 at different tumor grades is still elusive. Here, to investigate the mechanism underlying this correlation and identify the zinc transporter proteins as progression markers, we examined the changes in the protein expression pattern of zinc transporters depending on the HCC grade progression. First, we used both GEPIA web page and TCGA database to analyze the expression profile of 24 zinc transporters in hepatocellular cancer and normal tissue samples. RNA-Seq data of 50 controls and 371 patients with HCC from TCGA (https://tcga-data.nci.nih.gov/tcga/) (Weinstein et al., 2013) were taken using TCGAbiolinks (Colaprico et al., 2016) based on the R software (24) (v 4.1.0). According to data from both analyses, ZIP14 and ZIP5 transporters were found as common differentially expressed genes. However, these databases do not provide information on the transcriptional alterations during tumor progression. Due to this reason, in addition to ZIP5 and ZIP14, we have also examined ZnT1 (Zhu et al., 2021), ZnT7 (Tepaamorndech, Huang & Kirschke, 2011), ZIP7 (Taylor et al., 2008) transporters which have been previously reported as highly correlated with tumor progression.

This report establishes for the first time the relationship between the expression pattern of ZIP14, ZnT1 and ZIP5 and tumor grade. Our results provide new molecular insight into ZnT1, ZIP14 and ZIP5 mediated regulation of cellular zinc homeostasis, and indicate that zinc transporters might be important factors and events in HCC malignancy, which can lead to the development of early biomarkers.

## Material and Methods

### Ethical approval

This research project was approved by the Ankara University Human Ethics Committee on 11 of July 2018 (approval number: 10-675-18). We received written informed consent from the patients who participated in this study.

### Tissue specimens

We retrospectively and prospectively collected the data of 16 patients who underwent hepatic surgical resection and at Ankara University Ibn-i Sina Hospital (Ankara, Turkey). Formalin-fixed, paraffin-embedded (FFPE) tissues from patients with HCC were selected randomly from the archives of Ankara University Department of Pathology. Informed consent was obtained from all patients before the study was initiated with the approval of the Ankara University Human Ethics Committee in accordance with the Declaration of Helsinki. The tissues were assigned according to tumor grade. Out of 16 cases of HCC tissues five of them were graded as well differentiated (Grade I) HCC cases; seven of them were graded as moderately differentiated (Grade II) HCC cases and four of them were graded as poor differentiated (Grade III) HCC cases. Three cirrhotic surgically resected specimens which had adjacent non-neoplastic liver tissues were used as case-control.

### Immunohistochemical procedure

To estimate the correlations of zinc transporters and HCC prognosis immunohistochemical staining was performed in liver resection samples. Previously characterized HCC and cirrhotic FFPE tissue blocks were cut at 4 µm and dried for 1 h at 60 °C. Immunohistochemistry (IHC) staining was applied to tissue sections using fully automated Bond-Max IHC system (Leica). Subsequently, the slides were stained with antibodies against ZnT1 (MBS3013756), ZnT7 (MBS7605982), ZIP7 (MBS8525931), ZIP5 (MBS9412273) and ZIP14 (MBS9404998). All primary antibodies were purchased from MyBiosource. The sections were also counterstained with Mayer’s hematoxylin&eosin and cover slipped with mounting media (Thermo).

### Evaluation of IHC-positively stained tissues

Protein expressions on immunohistochemically stained tissues were semi-quantitatively evaluated with the Histological Score (H-Score) system. The percentage of the stained area was measured by NIH-ImageJ Fiji software (Fuhrich, Lessey & Savaris, 2013; Crowe & Yue, 2019). For the calculation of the H-Score, the percentage of positive area results was multiplied with “intensity of staining”. Intensity is categorized as weak, moderate and strong and signed with numeric values from 1 to 3, respectively (weak:1, moderate:2, strong:3). H-score = 1 × (% of faintly stained tumor area); 2 × (% of moderately stained tumor area); 3 × (% of strongly stained tumor area) (Fedchenko & Reifenrath, 2014). Thus, the H-score ranged from 0 to 300. Tissues with 10% of IHC positive tumor area were undertaken to determine. The intensity of the staining was scored by the pathologist.

### Gene expression profiling analysis

Cancer-specific webpage GEPIA (http://gepia.cancer-pku.cn/) and TCGA database to analyze the expression profile of 24 zinc transporters in hepatocellular cancer and normal tissue samples. According to data from GEPIA, 2206 genes were detected as differentially expressed in HCC. In addition to GEPIA, the clinical and RNA-Seq data of 50 normal and 369 patients with liver hepatocellular cancer (LIHC) from TCGA (https://tcga-data.nci.nih.gov/tcga/) (Weinstein et al., 2013) were retrieved using the Bioconductor (Gentleman et al., 2004) packages TCGAbiolinks (Colaprico et al., 2016) based on the R software (24) (v 4.1.0). Differential expression (DE) analysis comparing primary solid tumor samples against solid tissue normal was performed using the DESeq2 R package (Love, Huber & Anders, 2014) based on the negative binomial distribution after filtering for protein-coding genes in the count matrices using the useMart function provided by biomaRt package (Durinck et al., 2005). Differentially expressed transcripts were defined as those that satisfied two criteria: —log_2_(fold-change) —>1 and *p* < 0.05 after the Benjamini–Hochberg correction. The volcano plot (Fig. S1) highlighting the differentially expressed SLC39A and SLC30A family genes was generated using the EnhancedVolcano package (Blighe et al., 2021) in R. The raw numeric data obtained from each analysis was attached as Supplementary Material.

### Cell culture

Hep40 (well-differentiated) and Snu398 (poor differentiated) HCC cell lines were incubated in DMEM high glucose media containing 1% penicillin-streptomycin and 10% fetal bovine serum. The cells were passaged when reached to almost 80–90% confluency by tyripsinization. The human HCC cell lines Snu398 and Hep40 were obtained from ATCC (Manassas, VA).

### Protein detection by Western blotting

Total proteins from Hep40 and Snu398 cells were isolated as previously described (Bitirim, Ozer & Akcali, 2021). Protein concentration was determined by performing BCA assay (Thermo). Immunoblotting was performed using the monoclonal antibody against either ZIP14 (MyBiosource; MBS9404998) and ZnT1 antibody (MyBiosource; MBS3013756) at 1:300 concentration. Beta actin antibody (Santa Cruz; sc47778) was used as house-keeping control. Membranes were incubated in antibody solution at the concentration for overnight and 4 °C. The secondary antibody anti-Rabbit (Cell Signaling; 7074) anti-Goat (Santa Cruz; sc2354) was applied 1:2500 concentration for 1 h in blocking solution. ECL solution (Advansta) was used for chemiluminescence detection. The membranes were visualized by ChemiDoc gel imaging system (Bio-Rad, USA). Band intensity quantification was performed by ImageJ software (NIH).

### Intracellular free zinc measurement with flow cytometry in Hep40 and Snu398 cells

To measure the basal (resting) level of intracellular free zinc in Hep40 and Snu398 cells, we used zinc sensitive fluorescence dye-loaded cells, using non-ratiometric FluoZin-3 (3 µM FluoZin-3 AM) for flow cytometry (Acea, Novocyte). The cells were trypsinized from culture plate. Following washing and centrifugation steps, the pellet was resuspended in buffer which contains sodium azide and PBS. The 3 µM FluoZin-3 AM dye was added to this buffer-cell mixture and incubate for 30 min at room temperature. The Zn^2+^/Pyr concentration (10 µM) to obtain maximum Zn^2+^ saturation has been decided according to literature (Taylor et al., 2012) and our previous studies (Bitirim, Tuncay & Turan, 2018; Olgar et al., 2018). Fluorescence intensities were acquired as previously described (Bitirim, Tuncay & Turan, 2018). Intracellular free zinc density was calculated as fold change according to equation below (Bitirim, Tuncay & Turan, 2018):

\begin{eqnarray*}[Z{n}^{+2}]=Kd\times [(F-Fmin)/(Fmax-F)]. \end{eqnarray*}

### Statistical analysis

Groups were tested and compared using by unpaired Student’s *t*-test. The values from tissue sections were expressed as the means ± SD and from flow cytometry analysis were expressed as the means ± SD (*N* = 3). *p* < 0.05 was chosen statistically significant.

## Results

### Association of expression patterns of ZnT1 and ZIP14 and HCC prognosis

**Figure 2:**
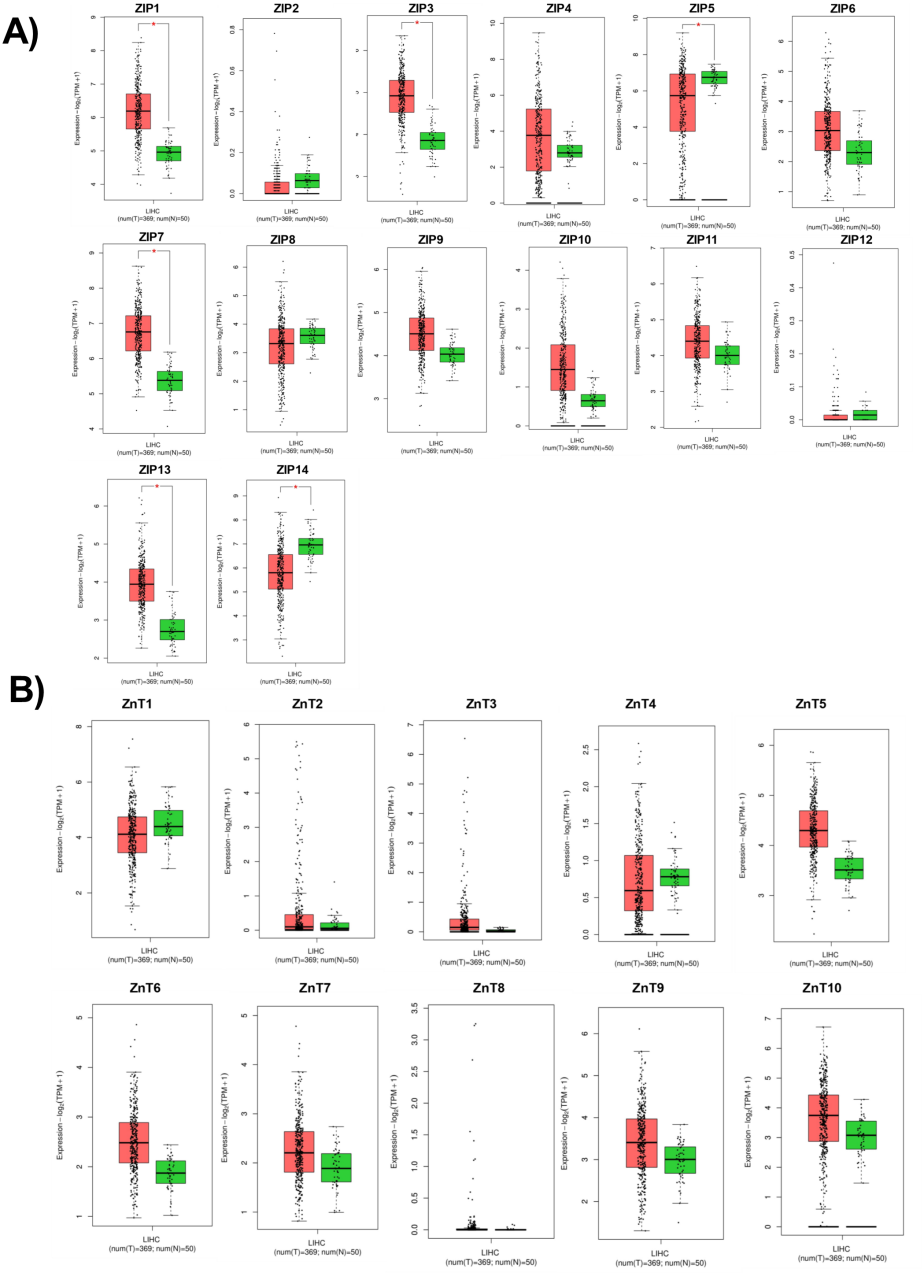
Cancer-specific webpage GEPIA (http://gepia.cancer-pku.cn/) was used to analyze the expression profile of 24 zinc transporters in 369 of hepatocellular cancer and 50 of normal patients. Analysis of RNA expression of (A) ZIP and (B) ZnT proteins in control (N =normal) and HCC tumor (T) patients. LIHC, Liver hepatocellular carcinoma. An asterisk (*) indicates *p* < 0, 05.

**Figure 3:**
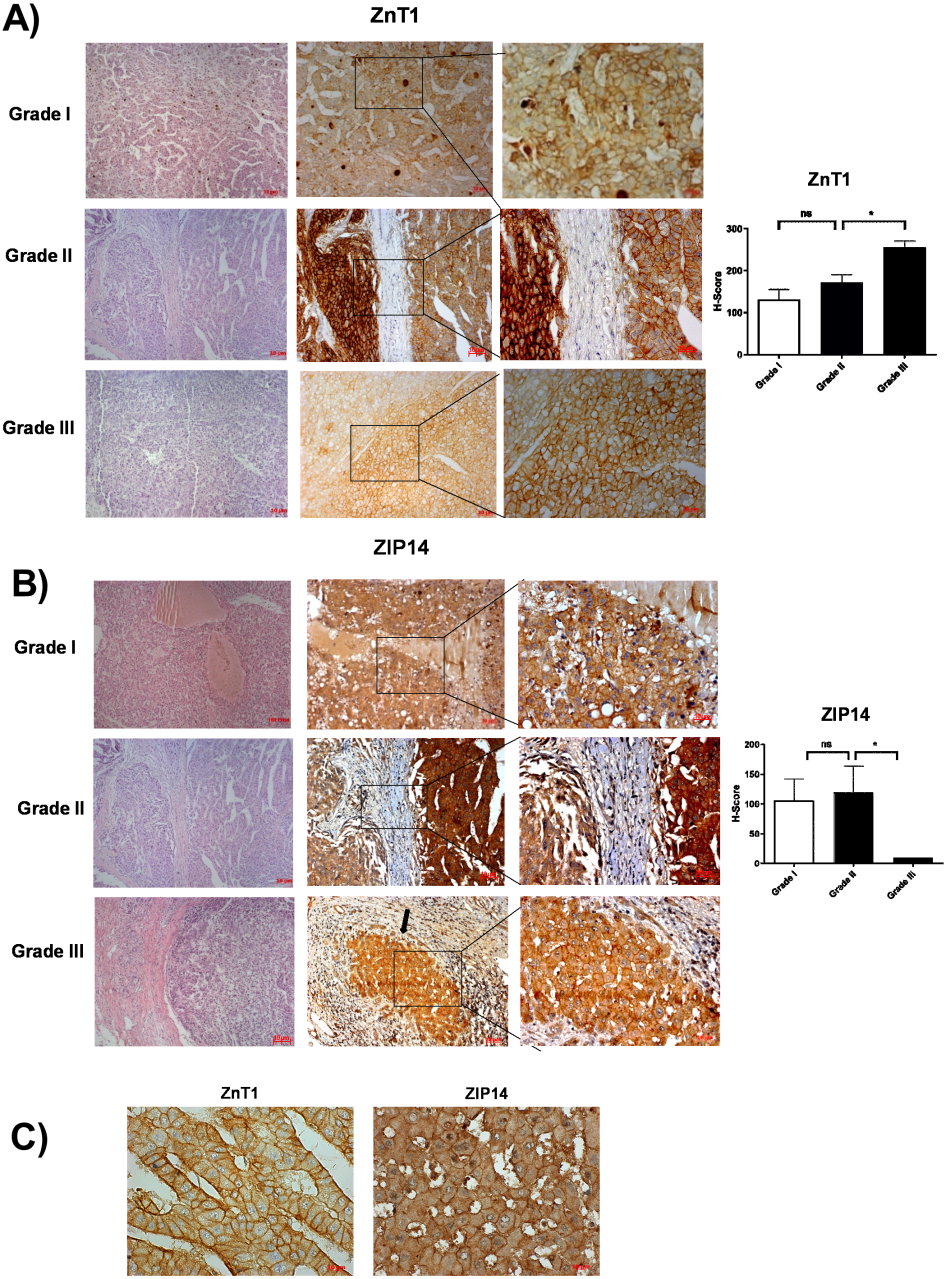
The evaluation of ZnT1 and ZIP14 protein expressions in Grade I, Grade II and Grade III HCC tissues by IHC. The representative images of H&E staining (magnification: 20X) and IHC staining (magnification: 20X and 40X) of ZnT1 (A) and ZIP 14 (B) in HCC tissues. Arrow indicates cirrhotic region. (C) Membrane staining of ZnT1 and ZIP14 (magnification: 63X). Data are expressed as the mean ± standard deviation. An asterisk (*) indicates *p* < 0.05. ns: not significant (*n* = 5 (grade I), *n* = 7 (grade II) *n* = 4 (grade III) HCC cases).

According to RNA expression data from GEPIA; ZIP1, ZIP3, ZIP5, ZIP7, ZIP13 and ZIP14 (Fig. 2A) were detected as differentially expressed in HCC. Although, it was observed that none of the ZnT genes are differentially expressed in HCC comparing to normal group (Fig. 2B). In addition, according to RNA-seq analysis from TCGA database; ZIP14, ZIP5, ZIP2, ZnT2, ZnT3, ZnT8 (Fig. S1) were detected as differentially expressed genes in HCC. ZIP14 and ZIP5 transporters were found as common differentially expressed genes from both bioinformatic analyses. Here we also investigated ZnT1, ZnT7 and ZIP7 transporters which have a role in tumor progression. Among 5 given zinc transporter proteins, no significant change was observed in the expression of ZnT1, ZnT7 and ZIP7 within different tumor grades (Fig. S2). The significant change in protein staining was observed only in ZnT1 and ZIP14 transporters. Although no significant increase was observed in the ZnT1 staining between Grade I (average H-score 131) and Grade II (average H-score 171,25) HCC tissues, the staining significantly stronger in Grade III (average H-score 255) comparing to Grade I and II HCC samples (Fig. 3A). In Grade I (average H-score 106) and Grade II (average H-score 119,28) HCC tissues, ZIP14 showed similar staining prevalence and intensity. But in Grade III HCCs, ZIP14 staining decreased almost 10-fold (average H-score 10) (Fig. 3B). ZnT1 and ZIP14 facilitates exporting of cytosolic zinc to the extracellular space and localizes predominantly in the cell membrane. Our IHC results showed complete membranous staining of ZnT1 and ZIP14 in the hepatocytes and epithelium (Fig. 3C). In addition, while ZIP14 staining was almost no detectable in Grade III tissue, it was observed in the cirrhotic region of the same tissue. These results demonstrated that the expression pattern of ZnT1 and ZIP14 zinc transporters in hepatocytes are correlated with hepatic tumor progression. This correlation may major factor of zinc depletion which occurs in HCC. H-scores of ZnT1 and ZIP14 staining is given in Table 1.

### Differential expression of ZIP5 and ZnT1 in cirrhotic liver and HCC

**Figure 4:**
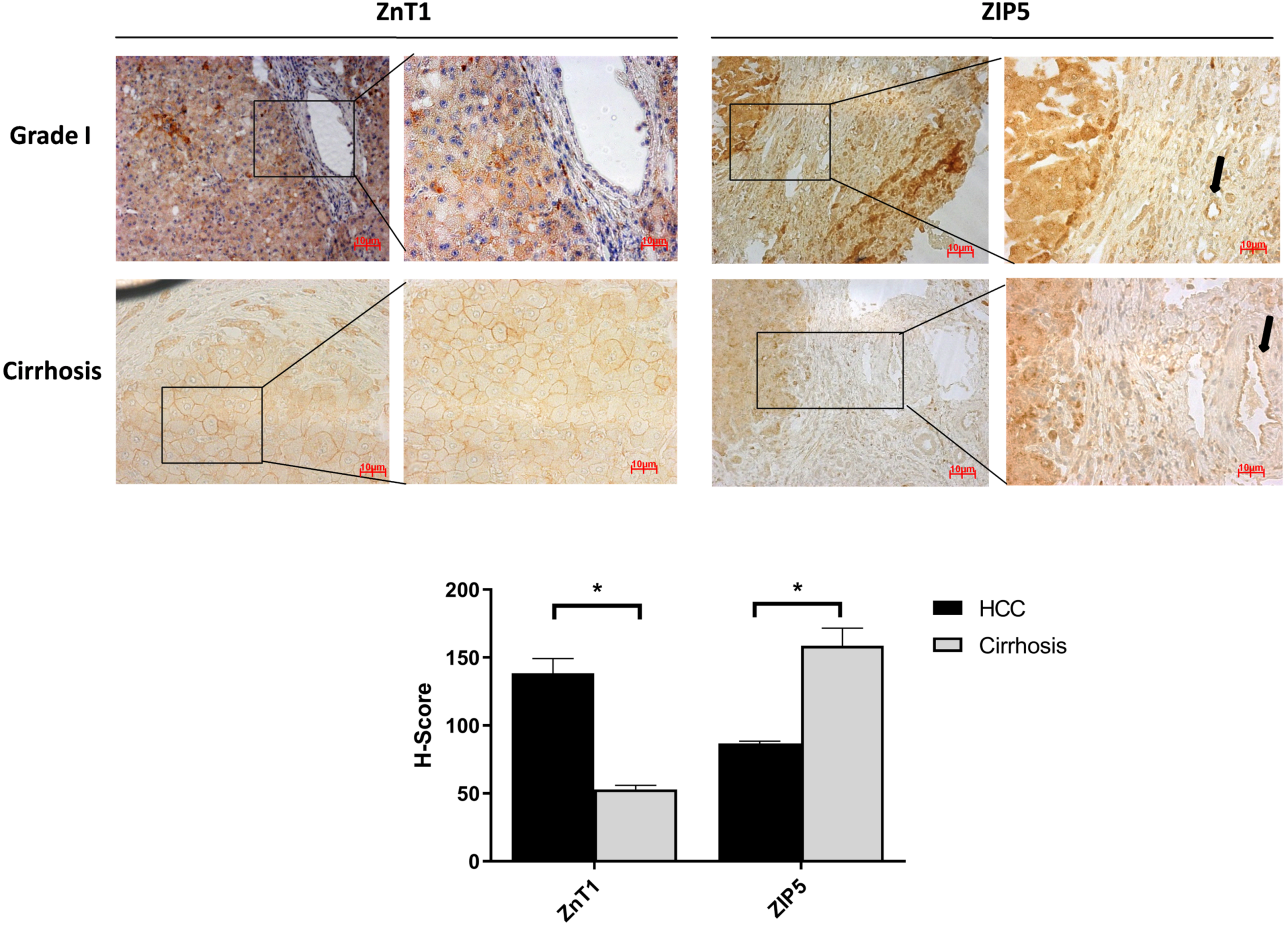
The evaluation of Znt1 and Zip5 protein expressions in cirrhotic and Grade I HCC tissue. The representative images of IHC staining (magnification, 20X and 40X) of ZnT1 and ZIP5. Arrow indicates vein. Data are expressed as the mean ± standard deviation. * indicates *p* < 0.05. ns: not significant. (*n* = 5 (grade I) HCC cases; *n* = 3 cirrhotic cases).

It is well known that progression of the cirrhosis leads to the development of HCC and zinc deficiency commonly is observed in patients with cirrhosis (Costello & Franklin, 2014). Due to this reason, the identification of proteins that may facilitate the transition from cirrhosis to HCC could be very important and useful for diagnosis. Among the other zinc transporter proteins, only ZIP5 and ZnT1 have shown significant and consistent changes within the cirrhotic tissue and Grade I HCC (Fig. 4). The granular ZIP5 staining was mostly observed in hepatocytes and endothelial cells of veins. ZIP5 staining was detected almost 2-fold higher in cirrhosis than HCC. On the contrary, ZnT1 staining was observed almost 3-fold lower in cirrhosis comparing to HCC. Moreover, ZIP14 protein expression has not shown a significant alteration between cirrhotic tissue and Grade I HCC (Fig. S3) while demonstrating a drastic reduction in HCC grade III.

### Intracellular zinc level in well and poor-differentiated HCC cells

**Figure 5:**
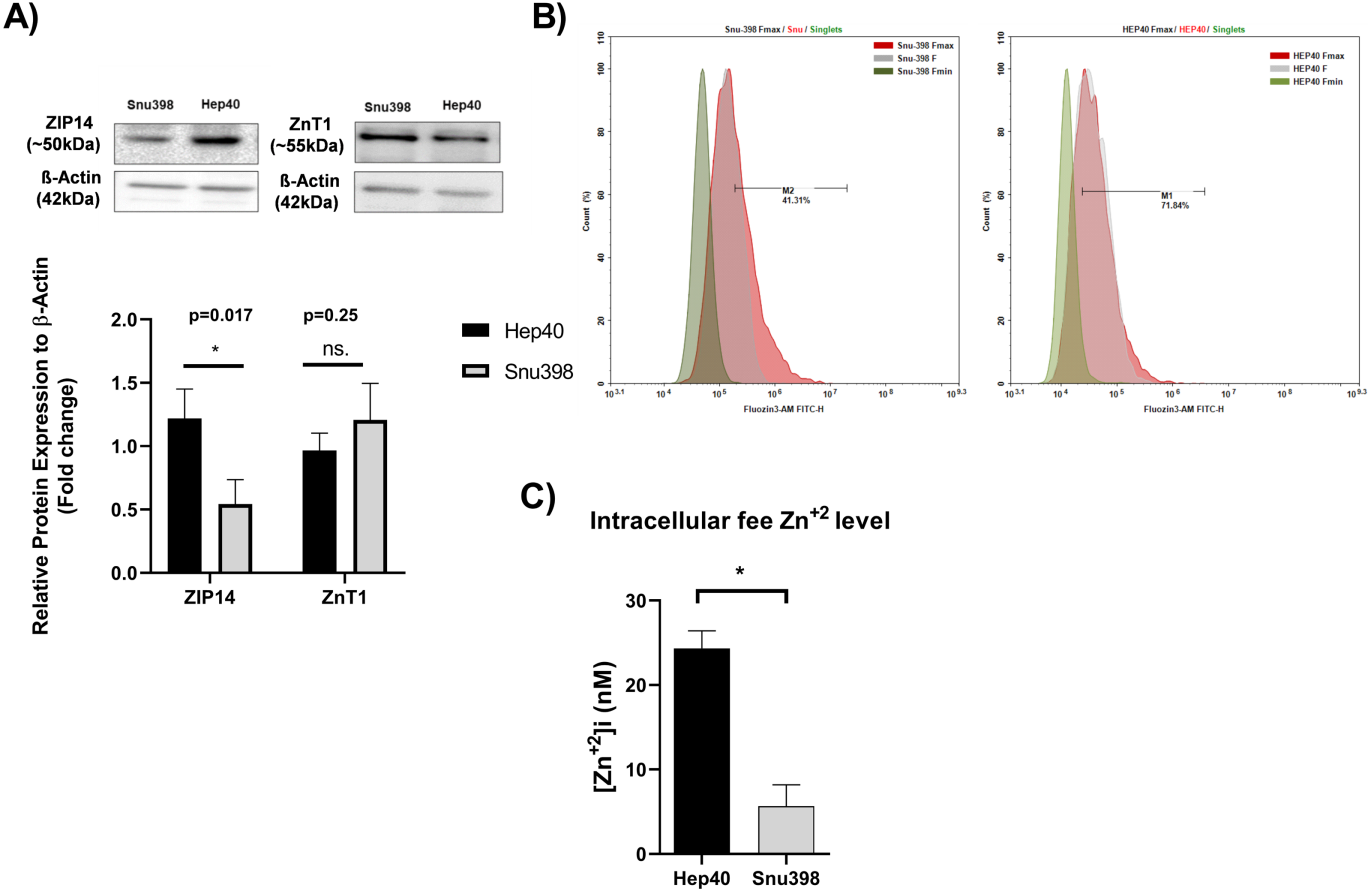
Intracellular free zinc measurement with flow cytometry in Hep40 and Snu398 cells. ZnT1 and ZIP14 proteins were detected in Hep40 and Snu398 cells by western blotting (A). The representative figure shows the flow cytometry analysis of basal (steady-state) (F), minium (Fmin) and maximum (Fmax) level of intracellular free zinc in Hep40 and Snu398 using non-ratiometric FluoZin-3 (3 µM FluoZin-3 AM) dye (B). Intracellular free zinc density was calculated as nM (C). Data are expressed as the mean ± standard deviation (*n* = 3). An asterisk (*) indicates *p* < 0.05. ns, not significant. M1 and M2 shows the percentage of FluoZin-3 positive cells. FITC indicates the label of FluoZin-3.

Measuring the tissue zinc level in “paraffin-embedded tissue sections” is not possible (Franklin, Levy & Costello, 2012; Costello & Franklin, 2014). In addition, zinc ions are present in the cells as “free” (or labile) intracellular form or bound form. The tissue zinc staining procedures allow the detection of both the intracellular free zinc and bound zinc ions. Free zinc ions can act as both second messengers in various signaling pathways or co-factor in proteins which include zinc-binding motif (Crowe & Yue, 2019). The similarity in the distinct expression pattern of clinically relevant metabolic genes between HCC cell lines and liver tumor tissues enable them as necessary source for basic and translational research on HCC.Due to this reason, we investigated the level of intracellular free zinc level Zn^2+^ ([Zn^2+^] (i) in different HCC cell lines originated tumor tissues at opposite grades Hep40 and Snu398 cell lines which represent Grade I and Grade III liver tumor specimens, respectively (Yuzugullu et al., 2009). The concentration of intracellular free zinc level Zn^2+^ was measured using FluoZin-3 staining (3 µM FluoZin-3 AM). Before the zinc measurement, to support the consistency between *in vitro* and *in vivo* experiments, we verified the presence of the ZIP14 and ZnT1 transporter proteins in the given HCC cell lines *via* western blotting (Fig. 5A). ZIP14 protein expression significantly increase in Hep40 cells, while ZnT1 tends to decrease. This result seems to be correlated with our IHC data obtained from liver tissue sections. The difference between Fmax and basal F value was higher in Hep40 cells which indicates the lower intracellular free zinc level of Hep40 compared to Snu398 cells (Fig. 5B). According to [Zn^2+^] i calculation, the [Zn^2+^] i decreased almost 9-fold in Snu398 HCC cells comparing to Hep40 HCC cells (Fig. 5C). This data revealed that intracellular zinc depletion may correlate with tumor progression in liver.

**Table 1:** H-scores of ZnT1 and ZIP14 stainings of surgically resected liver tissues from 16 HCC patients at Grade I, Grade II and Grade III.

		**ZnT1**			**ZIP14**		
** **	**#ID**	**Staining area (%)**	**Intensity of staining**	**H-Score**	**Staining area (%)**	**Intensity of staining**	**H-Score**
** GRADE I**	1	%90	1	90	%10	1	10
	2	%90	1	90	%30	1	30
	3	%95	2	190	%70	2	140
	4	%95	1	95	%95	2	190
	5	%95	2	190	%80	2	160
** GRADE II**	6	%80	2	160	%70	2	140
	7	%90	3	270	%10	1	10
	8	%80	1	80	%90	3	270
	9	%75	2	150	%70	1	70
	10	%70	2	140	%50	1	50
	11	%95	2	190	%10	1	10
	12	%95	2	190	%95	3	285
** GRADE III**	13	%80	3	240	%30	1	30
	14	%80	3	240	%10	1	10
	15	%100	3	300	N/A	0	0
	16	%80	3	240	%10	1	10

## Discussion

According to World Health Organization (WHO), zinc deficiency is one of the most important risk factors for morbidity and mortality in developing countries (Ollig et al., 2019). Hence, maintaining zinc homeostasis is vital for liver and liver ailments that may be influenced by zinc deficiency. Since intracellular zinc concentration levels play an important role in various carcinomas (Xu et al., 2014), it has been hypothesized that these changes in zinc levels may contribute to the advancement of tumors by influencing a vast variety of molecular structures, such as receptors, kinases, caspases, phosphatases and various transcriptional factors. The clinical evidences also demonstrated that Zn concentration in HCC tissues was lower compared to the surrounding hepatic parenchymal cells (Himoto & Masaki, 2018; Fang et al., 2019). However, few reports have described in detail the correlation of zinc level in early HCC patients with prognosis and the underlying mechanism of this “signature” clinical characteristic has been largely ignored (Hiraoka et al., 2020). This has impeded the potential application of the zinc relationship for a therapeutic approach and biomarkers for the identification of early malignancy and risks. Toward this, in the present study we have demonstrated the association of changes in the expression level of zinc transporter proteins with HCC prognosis and showed that the depletion of intracellular free zinc level may correlate with the aggression of tumor.

The upregulation of ZnT1 in HepG2 cells has been shown but there was no evidence of differential expression of ZnT1 within the different grades of HCC (Hiraoka et al., 2020). Our data revealed that the staining of ZnT1 transporter protein was stronger in Grade III comparing to Grade I and II HCC tissues. Notably, we observed that ZnT1 staining was higher in HCC tumor tissue comparing to cirrhotic tissues. In addition to the association of increased level of ZnT1 with tumor aggression, ZIP14 transporter protein staining significantly decreases in Grade III HCCs comparing to Grade I and Grade II. Our results have suggested the possibility that ZnT1 and ZIP14 may have reciprocal roles in zinc transport across the cell membrane and synergistically contribute zinc depletion in HCC. This depletion may correlate with increasing tumor grade. Of note, we did not observe the tumor grade-dependent change in ZnT7, ZIP7 or ZIP5 staining in HCC tissues (Fig. S2). Downregulation of ZIP5 at the gene and protein level in the liver upon chronic ethanol exposure was previously reported (Sun et al., 2014) .This data indicated that dysregulation of ZIP5 protein involves alcohol-induced hepatic zinc deficiency. Consistent with this result, we detected that ZIP5 staining markedly reduced in HCC tissue comparing to cirrhotic tissue. ZIP14 was also tested in cirrhotic tissue and no significant change was not observed. This data suggests that ZIP14 may contribute to the late tumorigenesis stage while ZIP5 protein expressions are related to the transition from liver cirrhosis to HCC. Taken together it can be suggested that each zinc protein may be responsible for the disruption of zinc homeostasis in different pathophysiological conditions.

Besides the role of ZIP14 in zinc transport, studies with Fe [non-transferrin-bound iron (NTBI)] and Mn^2+^ showed that ZIP14 transports these metals. Partial localization of ZIP14 with transferrin-bound iron (Fe-TF) was shown in HepG2 cells. Knocking down of ZIP14 in HepG2 cells, Fe accumulation reduces suggesting that ZIP14 contributes to the uptake of Fe-TF (Jenkitkasemwong et al., 2015). Several studies have demonstrated that these alterations of cellular iron metabolism are directly dependent on the action of oncogenes and tumor suppressors. Generally, it was considered that the high iron needs of tumor cells to sustain cell proliferation, the alterations of iron trafficking in cancer cells lead to iron acquisition (Manz et al., 2016). But for HCC, iron metabolism is a controversial issue, since it has been shown that iron supplementation promoted cell proliferation in HepG2 cells, representing the possible reason for carcinogenesis and neoplastic cell growth *via* influencing p53 ubiquitination pathway (Dongiovanni et al., 2010). The contribution of ZIP14 to Mn^2+^ transport system in rat hepatocytes was also speculated (Aydemir & Cousins, 2018). An inverse association between Mn^2+^ levels was demonstrated by meta-analysis (Chen et al., 2019). In conclusion, dysregulation of ZIP14 protein abundance may affect the tumor progression in the liver.

In addition to differential expression of zinc transporters, it is also necessary to examine the reduction of free zinc level which is correlated with metastasis capacity. Although the controversial data on serum and tissue zinc level, it has been reported through human studies that plasma zinc concentrations do not indicate total-body and tissue specific intracellular zinc stores reliably under all circumstances. It has been demonstrated that when the total body zinc content is reduced, fractional plasma zinc turnover rates are increased to compensate for tissue needs (Gibson et al., 2008). The total amount of zinc in plasma is <0.2% of the total body zinc content and the concentration of zinc in tissues such as muscle and liver are almost 50 times greater than in plasma. Due to this reason, small differences in uptake or release of zinc from these peripheral sites can have a profound effect on the plasma zinc concentration (Brown, 1998). Especially in patients with alcoholic hepatitis and cirrhosis, the difference between serum and tissue zinc levels was also evaluated. Both the clinical and animal studies suggest that serum zinc levels could be elevated at an early stage of alcoholic liver disease (Kang et al., 2009) but decreased at the advanced stage (Sun et al., 2014). Although plasma zinc level has been used as an indicator for dietary zinc deficiency (King et al., 2000), clinical and pre-clinical results suggest that plasma zinc level is not always a good indicator for assessing dietary zinc status and organ zinc status at different pathophysiological conditions. We postulated that the alteration in zinc transporter expression pattern may be responsible for the intracellular free zinc depletion which increases by the tumor aggression. Dithizone (DTZ) or Zinquin tissue staining is mostly used to detect bound and free zinc ions on frozen and tissue microarray sections. In addition, measuring “intracellular free zinc ions” is necessary to determine the impairment of zinc homeostasis in the cell (Ollig et al., 2019). Since both we have used paraffin tissue sections from patients and aimed to determine the change in free zinc level, we have measured the free zinc level *in vitro* using two different HCC cell lines.

Although cancer cell lines do not completely replicate physiological conditions of original tumors, numerous studies have demonstrated the resemblance of HCC cell lines to human liver tumors, in the expression of distinct metabolic targets. Most HCC cell lines have been characterized by multi-omic and proteomics techniques in terms of their mutation status, genome-wide mRNA and mi-RNA expression profiles and protein expression status (Hirschfield et al., 2018). HCC cell lines are identified as subgroups of epithelial-like and mesenchymal-like which based on the expression level of E-cadherin and Vimentin protein, similar to primary tumors (Fuchs et al., 2008). The cancer cell line encyclopedia (CCLE) (Barretina et al., 2012) and COSMIC (Forbes et al., 2015) projects have demonstrated the similarity in the expression pattern of clinically related metabolic genes between HCC cell lines and human liver tumors. In addition, proteomics and metabolomics analysis were integrated to enable a multi-level insight into the tumor molecular alterations depicted by HCC cell lines. Since the expression pattern of poor and well-differentiated HCC lines reflect HCC-tissue-derived metabolic genes, it was also suggested that altered targets in the cell lines could be relevant signatures in HCC (Nwosu et al., 2018).

As expected, intracellular free zinc level drastically decreased (almost 9-fold) in poor differentiated Snu398 HCC cells comparing to well-differentiated Hep40 HCC cells. Taken together we suggested that dysregulated expression of ZnT1 and ZIP14 may synergistically contribute zinc depletion which increases simultaneously with tumor progression in HCC.

## Conclusions

The present results highlight the role of zinc transporters; ZnT1, ZIP14 and ZIP5 in the regulation of cellular zinc homeostasis in HCC malignancy, suggesting that these zinc transporters may have the major roles in controlling intracellular zinc deficiency observed in HCC. Especially the dramatic difference in ZnT1 and ZIP14 staining between Grade II and Grade III suggested that these zinc transporters might be promising biomarkers that should be considered in HCC progression. The current study provides solid evidence that there is a remarkable correlation between zinc transporters and tumor prognosis.

## Supplemental Information

10.7717/peerj.12314/supp-1 Supplemental Information 1 Raw data for Figs. 2 and 3

10.7717/peerj.12314/supp-2 Supplemental Information 2 Raw data for Fig. 4C

10.7717/peerj.12314/supp-3 Supplemental Information 3 Raw data for Fig. 4A

10.7717/peerj.12314/supp-4 Supplemental Information 4 GEPIA AND RNA-seq analysis from TCGA- R code

10.7717/peerj.12314/supp-5 Supplemental Information 5 Volcano plot Volcano plot representation of DE analysis of genes in LIHC based on TCGA dataset. Gold points mark the genes with significantly increased or decreased expression (pCutoff = 1e−05). The *x*-axis shows log_2_fold-changes in expression and the *y*-axis the − log10 *P* values.

10.7717/peerj.12314/supp-6 Supplemental Information 6 The evaluation of ZnT7, ZIP7 and ZIP5 protein expression The evaluation of ZnT7, ZIP7 and ZIP5 protein expression in Grade I, Grade II and Grade III HCC tissues by IHC. IHC staining (magnification: 20X and 40X) in HCC tissues. Data are expressed as the mean ± standard deviation. * indicates *p* < 0.05. ns: not significant

10.7717/peerj.12314/supp-7 Supplemental Information 7 The evaluation of ZIP14 protein expressions in cirrhotic and Grade I HCC tissue The evaluation of ZIP14 protein expressions in cirrhotic and Grade I HCC tissue. Representative images of IHC staining (magnification, 20X and 40X) of ZIP14. Data are expressed as the mean ± standard deviation. * indicates *p* < 0.05. ns, not significant.

## Additional Information and Declarations

### Data Availability

The following information was supplied regarding data availability: The raw data is available in the Supplemental Files.

## References

[ref-1] Aydemir TB, Cousins RJ (2018). The multiple faces of the metal transporter ZIP14 (SLC39A14). The Journal of Nutrition.

[ref-2] Barretina J, Caponigro G, Stransky N, Venkatesan K, Margolin AA, Kim S, Wilson CJ, Lehár J, Kryukov GV, Sonkin D, Reddy A, Liu M, Murray L, Berger MF, Monahan JE, Morais P, Meltzer J, Korejwa A, Jané-Valbuena J, Mapa FA, Thibault J, Bric-Furlong E, Raman P, Shipway A, Engels IH, Cheng J, Yu GK, Yu J, Aspesi PJ, De Silva M, Jagtap K, Jones MD, Wang L, Hatton C, Palescandolo E, Gupta S, Mahan S, Sougnez C, Onofrio RC, Liefeld T, MacConaill L, Winckler W, Reich M, Li N, Mesirov JP, Gabriel SB, Getz G, Ardlie K, Chan V, Myer VE, Weber BL, Porter J, Warmuth M, Finan P, Harris JL, Meyerson M, Golub TR, Morrissey MP, Sellers WR, Schlegel R, Garraway LA (2012). The cancer cell line encyclopedia enables predictive modelling of anticancer drug sensitivity. Nature.

[ref-3] Bitirim CV, Ozer ZB, Akcali KC (2021). Estrogen receptor alpha regulates the expression of adipogenic genes genetically and epigenetically in rat bone marrow-derived mesenchymal stem cells. PeerJ.

[ref-4] Bitirim CV, Tuncay E, Turan B (2018). Demonstration of subcellular migration of CK2*α* localization from nucleus to sarco(endo)plasmic reticulum in mammalian cardiomyocytes under hyperglycemia. Molecular and Cellular Biochemistry.

[ref-5] Brown KH (1998). Effect of infections on plasma zinc concentration and implications for zinc status assessment in low-income countries. American Journal of Clinical Nutrition.

[ref-6] Chen X-B, Wei Y-H, Chen X-K, Zhong J, Zou Y-B, Nie J-Y (2019). Manganese levels and hepatocellular carcinoma. Medicine.

[ref-7] Colaprico A, Silva TC, Olsen C, Garofano L, Cava C, Garolini D, Sabedot TS, Malta TM, Pagnotta SM, Castiglioni I, Ceccarelli M, Bontempi G, Noushmehr H (2016). TCGAbiolinks: an R/Bioconductor package for integrative analysis of TCGA data. Nucleic Acids Research.

[ref-8] Costello LC, Franklin RB (2014). The status of zinc in the development of hepatocellular cancer: an important, but neglected, clinically established relationship. Cancer Biology and Therapy.

[ref-9] Crowe AR, Yue W (2019). Semi-quantitative determination of protein expression using immunohistochemistry staining and analysis: an integrated protocol. Bio-Protocol.

[ref-10] Dongiovanni P, Fracanzani AL, Cairo G, Megazzini CP, Gatti S, Rametta R, Fargion S, Valenti L (2010). Iron-dependent regulation of MDM2 influences p53 activity and hepatic carcinogenesis. American Journal of Pathology.

[ref-11] Durinck S, Moreau Y, Kasprzyk A, Davis S, De Moor B, Brazma A, Huber W (2005). BioMart and bioconductor: a powerful link between biological databases and microarray data analysis. Bioinformatics.

[ref-12] Fang A-P, Chen P-Y, Wang X-Y, Liu Z-Y, Zhang D-M, Luo Y, Liao G-C, Long J-A, Zhong R-H, Zhou Z-G, Xu Y-J, Xu X-J, Ling W-H, Chen M-S, Zhang Y-J, Zhu H-L (2019). Serum copper and zinc levels at diagnosis and hepatocellular carcinoma survival in the Guangdong Liver Cancer Cohort. International Journal of Cancer.

[ref-13] Fedchenko N, Reifenrath J (2014). Different approaches for interpretation and reporting of immunohistochemistry analysis results in the bone tissue - a review. Diagnostic Pathology.

[ref-14] Forbes SA, Beare D, Gunasekaran P, Leung K, Bindal N, Boutselakis H, Ding M, Bamford S, Cole C, Ward S, Kok CY, Jia M, De T, Teague JW, Stratton MR, McDermott U, Campbell PJ (2015). COSMIC: exploring the world’s knowledge of somatic mutations in human cancer. Nucleic Acids Research.

[ref-15] Franklin RB, Levy BA, Zou J, Hanna N, Desouki MM, Bagasra O, Johnson LA, Costello LC (2012). ZIP14 zinc transporter downregulation and zinc depletion in the development and progression of hepatocellular cancer. Journal of Gastrointestinal Cancer.

[ref-16] Fuchs BC, Fujii T, Dorfman JD, Goodwin JM, Zhu AX, Lanuti M, Tanabe KK (2008). Epithelial-to-mesenchymal transition and integrin-linked kinase mediate sensitivity to epidermal growth factor receptor inhibition in human hepatoma cells. Cancer Research.

[ref-17] Fuhrich DG, Lessey BA, Savaris RF (2013). Comparison of HSCORE assessment of endometrial beta3 integrin subunit expression with digital HSCORE using computerized image analysis (ImageJ). Analytical and Quantitative Cytopathology and Histopathology.

[ref-18] Gibson RS, Hess SY, Hotz C, Brown KH (2008). Indicators of zinc status at the population level: a review of the evidence. British Journal of Nutrition.

[ref-19] Grüngreiff K, Reinhold D, Wedemeyer H (2016). The role of zinc in liver cirrhosis. Annals of Hepatology.

[ref-20] Himoto T, Masaki T (2018). Associations between zinc deficiency and metabolic abnormalities in patients with chronic liver disease. Nutrients.

[ref-21] Hiraoka A, Nagamatsu K, Izumoto H, Adachi T, Yoshino T, Tsuruta M, Aibiki T, Okudaira T, Yamago H, Iwasaki R, Suga Y, Mori K, Miyata H, Tsubouchi E, Ninomiya T, Kawasaki H, Hirooka M, Matsuura B, Abe M, Hiasa Y, Michitaka K (2020). Zinc deficiency as an independent prognostic factor for patients with early hepatocellular carcinoma due to hepatitis virus. Hepatology Research.

[ref-22] Hirschfield H, Bian CB, Higashi T, Nakagawa S, Zeleke TZ, Nair VD, Fuchs BC, Hoshida Y (2018). In vitro modeling of hepatocellular carcinoma molecular subtypes for anti-cancer drug assessment. Experimental & Molecular Medicine.

[ref-23] Jenkitkasemwong S, Wang CY, Coffey R, Zhang W, Chan A, Biel T, Kim JS, Hojyo S, Fukada T, Knutson MD (2015). SLC39A14 is required for the development of hepatocellular iron overload in murine models of hereditary hemochromatosis. Cell Metabolism.

[ref-24] Kang X, Zhong W, Liu J, Song Z, McClain CJ, Kang YJ, Zhou Z (2009). Zinc supplementation reverses alcohol-induced steatosis in mice through reactivating hepatocyte nuclear factor-4 *α* and peroxisome proliferator-activated receptor-*α*. Hepatology.

[ref-25] Lichten LA, Cousins RJ (2009). Mammalian zinc transporters: nutritional and physiologic regulation. Annual Review of Nutrition.

[ref-26] Love MI, Huber W, Anders S (2014). Moderated estimation of fold change and dispersion for RNA-seq data with DESeq2. Genome Biology.

[ref-27] Manz DH, Blanchette NL, Paul BT, Torti FM, Torti SV (2016). Iron and cancer: recent insights. Annals of the New York Academy of Sciences.

[ref-28] Nwosu ZC, Battello N, Rothley M, Piorońska W, Sitek B, Ebert MP, Hofmann U, Sleeman J, Wölfl S, Meyer C, Megger DA, Dooley S (2018). Liver cancer cell lines distinctly mimic the metabolic gene expression pattern of the corresponding human tumours. Journal of Experimental & Clinical Cancer Research.

[ref-29] Olgar Y, Degirmenci S, Durak A, Billur D, Can B, Kayki-Mutlu G, Arioglu-Inan EE, Turan B (2018). Aging related functional and structural changes in the heart and aorta: MitoTEMPO improves aged-cardiovascular performance. Experimental Gerontology.

[ref-30] Ollig J, Kloubert V, Taylor KM, Rink L (2019). B cell activation and proliferation increase intracellular zinc levels. Journal of Nutritional Biochemistry.

[ref-31] Omran DA, Darweesh SK, Fouad H, Mahmoud M, Saif S, Fared A, Hassany M, Mobarak L, El-Tahawy MA, Yosry A (2017). Serum zinc deficiency and its relation to liver fibrosis in chronic HCV: a real-life egyptian study. Biological Trace Element Research.

[ref-32] Patel P, Bansal A (2021). Zinc level in hepatocellular carcinoma: a meta- analysis. Journal of Cancer Prevention & Current Research.

[ref-33] Portolani N, Coniglio A, Ghidoni S, Giovanelli M, Benetti A, Tiberio GAM, Giulini SM (2006). Early and late recurrence after liver resection for hepatocellular carcinoma: prognostic and therapeutic implications. Annals of Surgery.

[ref-34] Qian Y, Daza J, Itzel T, Betge J, Zhan T, Marmé F, Teufel A (2021). Prognostic cancer gene expression signatures: current status and challenges. Cell.

[ref-35] Siegel R, Naishadham D, Jemal A (2012). Cancer statistics, 2012. CA: A Cancer Journal for Clinicians.

[ref-36] Sun Q, Li Q, Zhong W, Zhang J, Sun X, Tan X, Yin X, Sun X, Zhang X, Zhou Z (2014). Dysregulation of hepatic zinc transporters in a mouse model of alcoholic liver disease. American Journal of Physiology - Gastrointestinal and Liver Physiology.

[ref-37] Taylor KM, Hiscox S, Nicholson RI, Hogstrand C (2012). Europe PMC Funders Group Protein kinase CK2 triggers cytosolic zinc signaling pathways by phosphorylation of zinc channel ZIP7.

[ref-38] Taylor KM, Vichova P, Jordan N, Hiscox S, Hendley R, Nicholson RI (2008). ZIP7-mediated intracellular zinc transport contributes to aberrant growth factor signaling in antihormone-resistant breast cancer cells. Endocrinology.

[ref-39] Tepaamorndech S, Huang L, Kirschke CP (2011). A null-mutation in the Znt7 gene accelerates prostate tumor formation in a transgenic adenocarcinoma mouse prostate model. Cancer Letters.

[ref-40] To PK, Do MH, Cho JH, Jung C (2020). Growth modulatory role of zinc in prostate cancer and application to cancer therapeutics. International Journal of Molecular Sciences.

[ref-41] Weinstein JN, Collisson EA, Mills GB, Shaw KRM, Ozenberger BA, Ellrott K, Shmulevich I, Sander C, Stuart JM (2013). The Cancer Genome Atlas Pan-Cancer analysis project. Nature Genetics.

[ref-42] Xu X, Guo HJ, Xie HY, Li J, Zhuang RZ, Ling Q, Zhou L, Wei XY, Liu ZK, Ding SM, Chen KJ, Xu ZY, Zheng SS (2014). ZIP4, a novel determinant of tumor invasion in hepatocellular carcinoma, contributes to tumor recurrence after liver transplantation. International Journal of Biological Sciences.

[ref-43] Yuzugullu H, Benhaj K, Ozturk N, Senturk S, Celik E, Toylu A, Tasdemir N, Yilmaz M, Erdal E, Akcali KC, Atabey N, Ozturk M (2009). Canonical Wnt signaling is antagonized by noncanonical Wnt5a in hepatocellular carcinoma cells. Molecular Cancer.

[ref-44] Zhu B, Huo R, Zhi Q, Zhan M, Chen X, Hua Z-C (2021). Increased expression of zinc transporter ZIP4, ZIP11, ZnT1, and ZnT6 predicts poor prognosis in pancreatic cancer. Journal of Trace Elements in Medicine and Biology.

