# Supplementary material for: The role of zinc transporter proteins as predictive and prognostic biomarkers of hepatocellular cancer
Source: PeerJ. 2021 Oct 15;9:e12314. doi: 10.7717/peerj.12314 (PMC8522644; doi:10.7717/peerj.12314)
Supplement: Supplemental Information 3 [file peerj-09-12314-s003.pptx]

## Slide 1
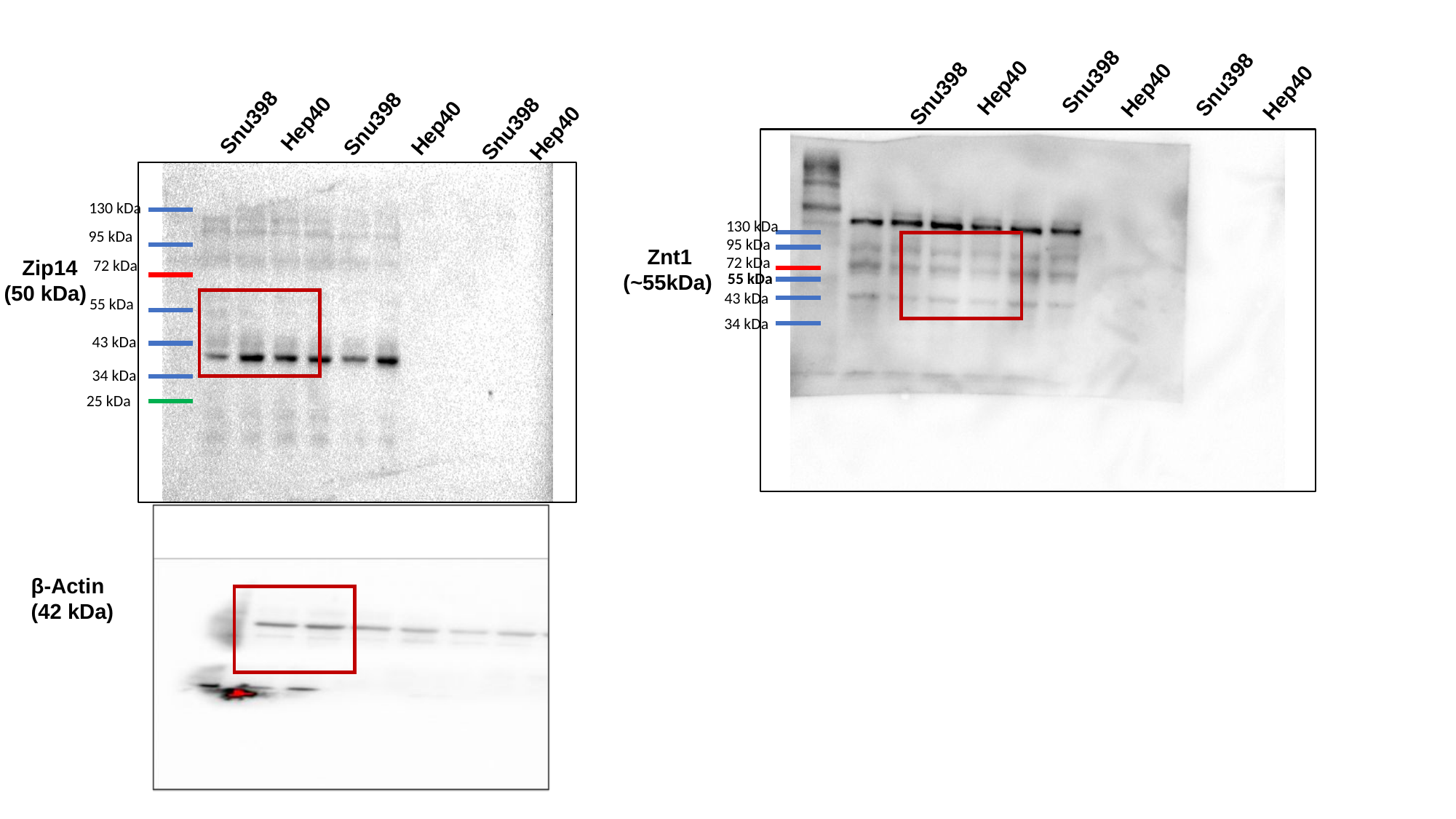

Hep40
Snu398
Hep40
Snu398
Hep40
Snu398
Hep40
Hep40
Snu398
Snu398
Hep40
Snu398
130 kDa
130 kDa
95 kDa
Hep40
Snu398
95 kDa
 Znt1
(~55kDa)
72 kDa
 Zip14
(50 kDa)
72 kDa
55 kDa
43 kDa
55 kDa
34 kDa
43 kDa
34 kDa
25 kDa
β-Actin
(42 kDa)
