# Supplementary figures and images for: The role of zinc transporter proteins as predictive and prognostic biomarkers of hepatocellular cancer
Source: PeerJ. 2021 Oct 15;9:e12314. doi: 10.7717/peerj.12314 (PMC8522644; doi:10.7717/peerj.12314)

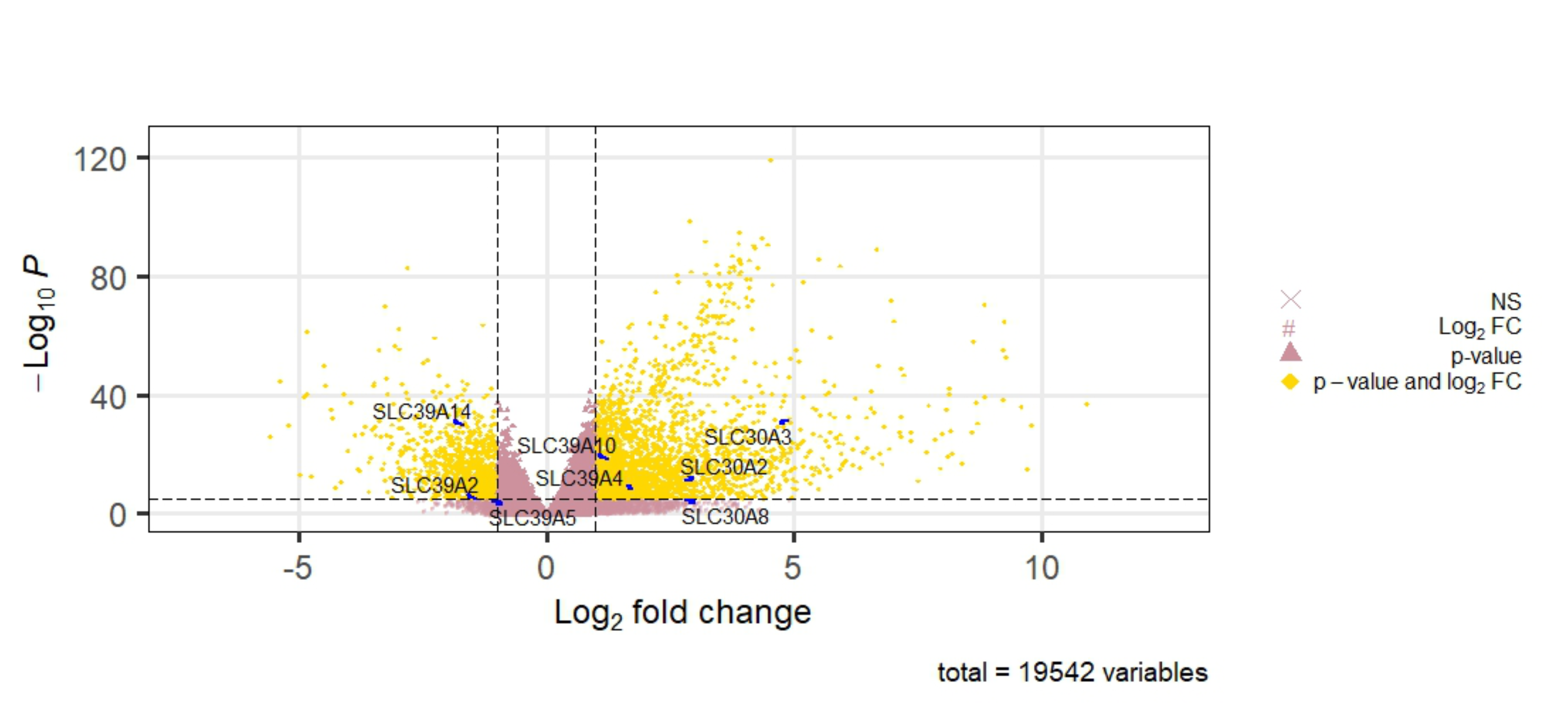

Supplement: Supplemental Information 5 — Volcano plot representation of DE analysis of genes in LIHC based on TCGA dataset. Gold points mark the genes with significantly increased or decreased expression (pCutoff = 1e−05). The x-axis shows log2fold-changes in expression and the y-axis the − log10 P values. [file peerj-09-12314-s005.png]

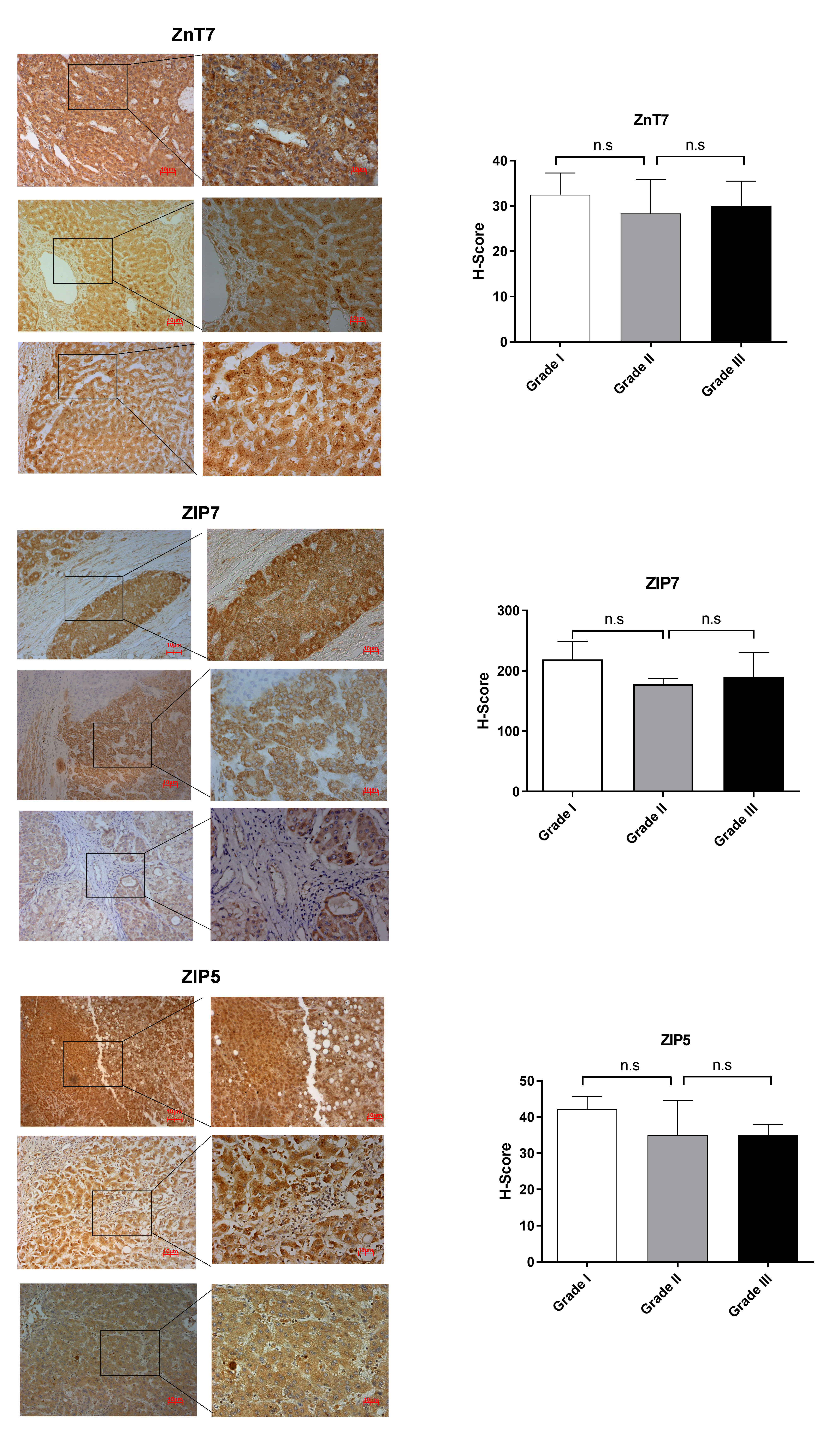

Supplement: Supplemental Information 6 — The evaluation of ZnT7, ZIP7 and ZIP5 protein expression in Grade I, Grade II and Grade III HCC tissues by IHC. IHC staining (magnification: 20X and 40X) in HCC tissues. Data are expressed as the mean ± standard deviation. * indicates p < 0.05. ns: not significant [file peerj-09-12314-s006.png]

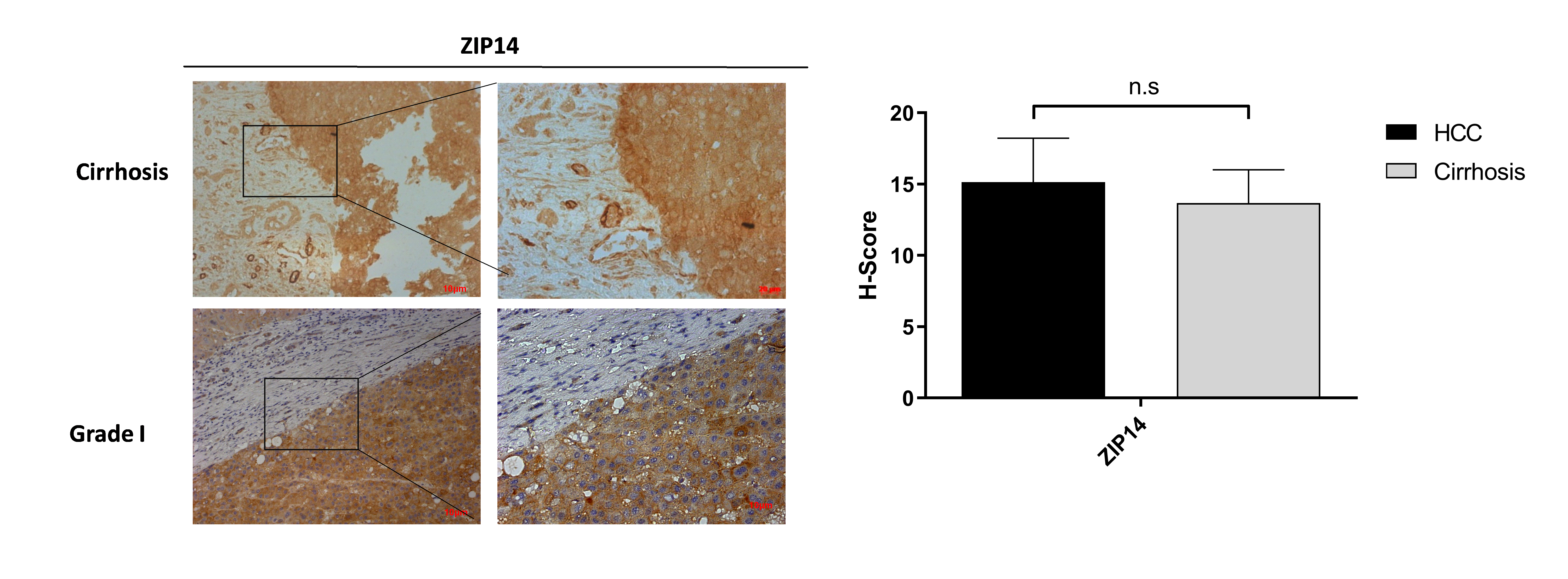

Supplement: Supplemental Information 7 — The evaluation of ZIP14 protein expressions in cirrhotic and Grade I HCC tissue. Representative images of IHC staining (magnification, 20X and 40X) of ZIP14. Data are expressed as the mean ± standard deviation. * indicates p < 0.05. ns, not significant. [file peerj-09-12314-s007.png]
